# Strength Characteristics, Ultrasonic Wave Velocity, and the Correlation between Them in Clay Bricks under Dry and Saturated Conditions

**DOI:** 10.3390/ma17061353

**Published:** 2024-03-15

**Authors:** Amin Jamshidi, Luís Sousa

**Affiliations:** 1 Department of Geology, Faculty of Basic Sciences, Lorestan University, Khorramabad 681151-44316, Iran; jamshidi.am@lu.ac.ir; 2 Department of Geology and Pole of Geosciences Center, University of Trás-os-Montes e Alto Douro, 5000-801 Vila Real, Portugal

**Keywords:** clay brick, predictive equation, saturation degree, strength characteristic, ultrasonic wave velocity

## Abstract

One of the methods used to discover the development of deterioration in bricks used as a construction material in a building is the monitoring of the bricks’ strength characteristics over time. However, measuring the strength characteristics of bricks used in a building requires sampling for performing laboratory tests, which is not possible in some cases. As an alternative, ultrasonic wave velocity can be a useful, nondestructive tool for the indirect assessment of the strength characteristics of the bricks. In the present study, six different samples of clay bricks before utilization as construction materials in buildings located in Khorramabad City (Lorestan Province, western Iran) were collected. The mineralogical composition of the samples was studied using X-ray diffraction (XRD) analysis. As one common physical characteristic of the construction materials, the porosity (n) of the samples was measured. Next, the strength characteristics, including uniaxial compressive strength (UCS), Brazilian tensile strength (BTS), and P-wave velocity (Vp), of the samples under dry and saturated conditions were determined. It was found that after the saturation of the samples, considerable decreases in the UCS and BTS and increases in the Vp occurred, respectively. By comparing the values of the UCS, BTS, and Vp of the samples under dry and saturated conditions, we found that the integrity loss for the UCS and BTS was higher than for the Vp. Results showed that the integrity loss of the UCS, BTS, and Vp was significantly affected by the n and clay mineral (CM) content of the samples. Considering the dry or saturated condition of the samples, there are good correlations with acceptable accuracy levels between the Vp and the UCS and BTS, with coefficients of determination (R^2^) varying from 0.95 to 0.98. Consequently, our findings showed that establishing UCS and BTS predictive equations for bricks before their use as a construction material can be a worthy, practical tool for monitoring the deterioration of bricks over time after their utilization in a building.

## 1. Introduction

**Figure 1:**
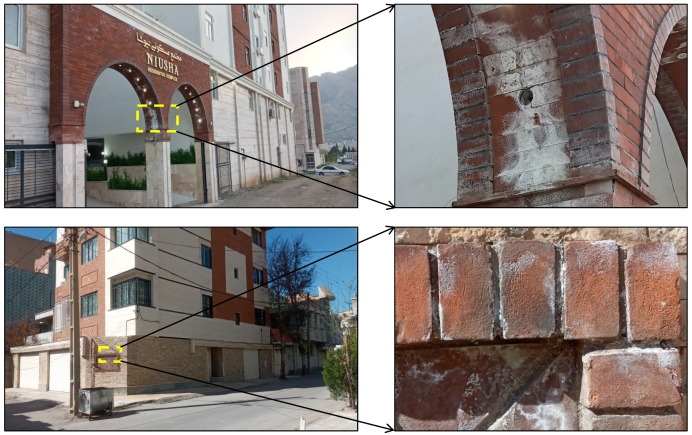
Examples of deterioration of bricks used as external cladding for a building (Khorramabad City, Lorestan Province, western Iran).

Clay brick is one of the most common construction materials used in buildings [1]. Under some climatic conditions, weathering processes such as freeze–thaw, salt crystallization, acid corrosion, wetting–drying, and groundwater erosion can lead to brick deterioration and, thus, damage to the building [2,3,4,5]. Figure 1 shows examples of the deterioration of bricks used as construction materials in buildings caused by the harmful effects of weathering processes such as salt crystallization. Efflorescence due to salt crystallization is among the most critical issues related to the aesthetic aspects of bricks used in modern and heritage buildings. In addition, salt crystallization action exerts stresses in the pores of the brick. When the induced stresses exceed the strength of the brick, new pores are created, and preexisting pores widen and deepen. Consequently, these changes affect the inherent characteristics of the brick and change them during its service life, thereby limiting its durability [5]. The first step to preventing the integrity loss of bricks subjected to weathering processes is to detect the beginning of deterioration and monitor its change intensity over time. This is a critical issue for preventive or restorative measures concerning brick deterioration under weathering processes a building is subjected to. Brick’s durability (i.e., its resistance to deterioration) against weathering processes is a function of various factors including its inherent characteristics, place of its use in a building, and the weathering process type [6]. Among brick’s inherent characteristics, those that are related to strength parameters [such as uniaxial compressive strength (UCS) and Brazilian tensile strength (BTS)] are very influential on the brick’s durability [1,7]. Therefore, any change in the strength characteristics can be considered evidence in discovering the occurrence of the brick’s deterioration. For this connection, one of the outstanding tools to detect the development of deterioration in brick over time is the monitoring of its UCS and BTS during various time periods. However, determining the UCS and BTS requires sampling from the bricks utilized in a building to perform relevant tests. In some cases, sampling is impossible, for example, when the brick size is small such that it does not allow for the preparation of the standard specimens for UCS and BTS tests, or when the sampling of the brick leads to visual damage to the building. Under these conditions, in situ measuring of P-wave velocity (Vp) on the brick used in the building can be a practical solution for the indirect assessment of UCS and BTS. Vp testing is nondestructive and easy to perform under both site and laboratory conditions [8,9]. This test, due to the portability, rapidity, and simplicity of its performance, and because it is low-cost and nondestructive, has been increasingly used by various researchers for a quick assessment of the UCS and BTS of construction materials [10,11,12,13]. One of the most important factors affecting UCS, BTS, and Vp, and thus correlations between them, is the saturation degree (Sr) of the construction materials [14,15,16,17,18]. The development of correlations under dry and saturated conditions may result in predictive equations with different accuracies to assess the UCS and BTS from the Vp. Failure to take the Sr into account when establishing correlation equations can lead to prediction errors of the UCS and BTS from the Vp and, as a result, inaccurate assessment of construction materials’ quality [19]. When the construction materials are porous, for example, clay brick, the Sr plays a more significant role in the predictive equations. Thus, Sr as a factor affecting the accuracy of predictive equations should be considered in the correlation analyses of UCS and BTS with Vp.

Although researchers have investigated the Sr effects on various characteristics of construction materials and also the correlation equations between them [18,20,21,22,23,24,25,26], in this regard, brick as one of the common construction materials used in buildings has been neglected in previous studies.

In the present study, by performing various laboratory tests on six different samples of clay bricks, three main goals were pursued: (i) investigating the Sr effect on the UCS, BTS, and Vp of the samples, (ii) investigating the role of n and clay minerals (CM) on the integrity loss of the UCS, BTS, and Vp of the samples due to their saturation, and (iii) developing the predictive equations for the UCS and BTS using the Vp under dry and saturated conditions of the samples.

## 2. Materials and Methods

**Figure 2:**
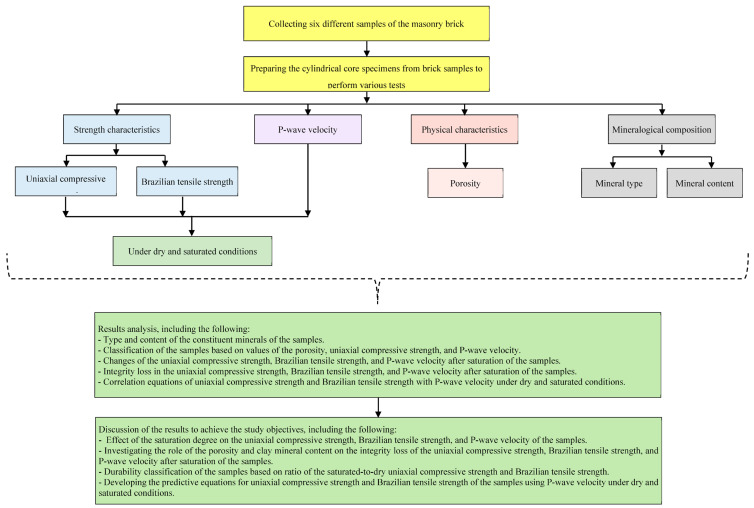
Flowchart of the study method.

Six different samples of clay bricks, which are widely used in some buildings located in Khorramabad City (Lorestan Province, western Iran) as cladding, were collected. Sampling of the bricks was performed before use them in buildings. The mineralogical composition of the samples was investigated using X-ray diffraction (XRD) analysis. Using a coring machine, cylindrical core specimens were prepared from brick samples for physical and strength tests that included n, UCS, BTS, and Vp. Specimens under dry and saturated conditions were tested, and their UCS, BTS, and Vp were measured. The data obtained from mineralogical investigations and physical and strength tests were analyzed to achieve the study objectives. In detail, the flowchart presented in Figure 2 shows the study method.

### 2.1. Mineralogical Composition

**Figure 3:**
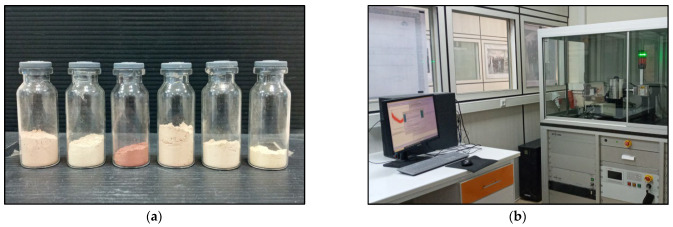
XRD analysis of the samples: (**a**) powder specimens prepared for analysis, and (**b**) X-ray diffractometer used in the present study.

The type and content of the constituent components of the samples were determined using XRD analysis. Among researchers, XRD analysis is one of the most widely used and prominent methods in mineralogical studies and provides valuable information regarding the type and content of the constituent minerals of construction materials [27,28]. In the present study, powder specimens from each brick sample were prepared and analyzed using an X-ray diffractometer (STOE model) with a copper (Cu) anode tube (Figure 3). Based on the XRD patterns obtained from the analyses, the type and content of the minerals that exist in the brick samples were investigated.

### 2.2. Physical Characteristics

**Figure 4:**
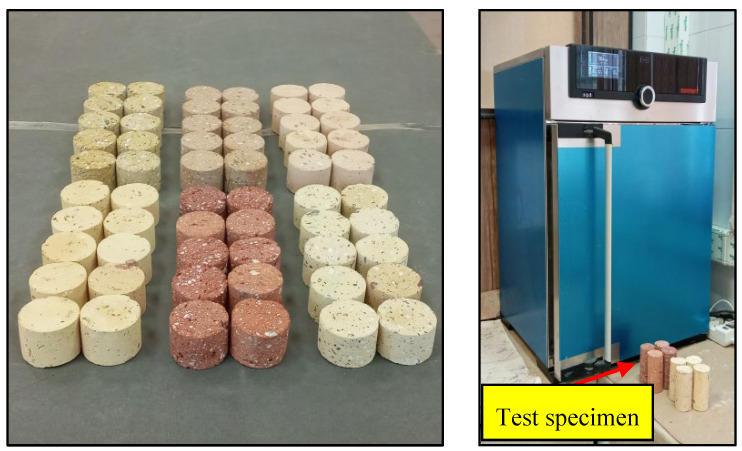

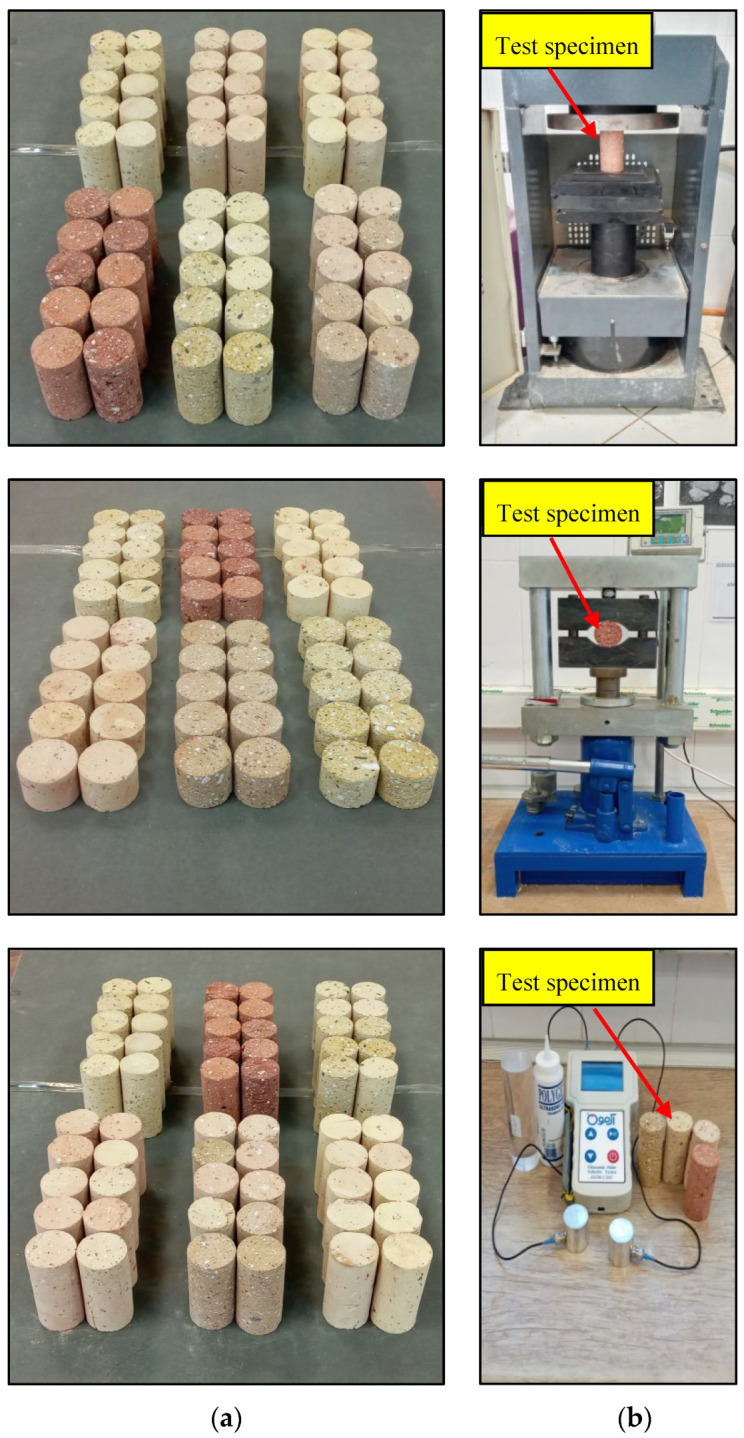
(**a**) Some of the specimens used for laboratory tests, and (**b**) devices to perform various tests.

The n as an index physical characteristic was determined for the brick samples in accordance with the ISRM [29]. This characteristic is among the critical parameters for assessment of construction material suitability for various applications in buildings [30,31]. For each brick sample, ten cylindrical core specimens with a diameter (D) and a thickness (T) of 44 and 30 mm, respectively, were used for the n tests (Figure 4). The porosity of the specimens was determined according to following equation:

(1) $$n=\frac{Vv}{V}\times 100$$

where Vv and V are pores and bulk volumes of the specimen, respectively, which were calculated using Equations (2) and (3), as follows:

(2) $$Vv=\frac{m_{sat}-m_{d}}{\rho_{w}}$$

where ρ_w_ is the water density, m_sat_ is the saturated mass of the specimen after being submerged in a bath filled with distilled water for 72 h, and m_d_ is the oven-dried mass of the specimen after being placed for 48 h in an oven at a temperature of 105 °C.

(3) $$V=\pi {\left(\frac{D}{2}\right)}^{2}T$$

where D and T are the diameter and thickness of the specimen.

### 2.3. Strength Characteristics

For each brick sample, two series of ten specimens were prepared, the first series for dry conditions and the second series for saturated conditions. The specimens were tested to determine their UCS following the method suggested by the ISRM [29]. Tests were performed on specimens with a diameter of 44 mm and a length-to-diameter ratio of 2. Some of the specimens used for the UCS tests are shown in Figure 4. Using the UCS device, loading was gradually applied to the specimens such that, finally, failure occurred in them. After recording the failure load (P_f_) and using the diameter of the specimen (D), the UCS was calculated from the following equation:

(4) $$UCS=\frac{P_{f}}{\pi {\left(\frac{D}{2}\right)}^{2}}$$

As recommended by the ISRM [29], BTS tests were carried out on specimens with a diameter (D) and thickness (T) of 44 and 30 mm, respectively. After placing the specimens in the BTS testing device, the load at a constant rate was applied to them such that failure occurred within a few minutes (Figure 4). Twenty specimens from each brick sample were tested under dry and saturated conditions, and their BTS was determined using Equation (5), as follows:

(5) $$BTS=\frac{2P_{f}}{\pi DT}$$

where P_f_ is the maximum recorded load in the moment of specimen failure.

### 2.4. P-Wave Velocity

Twenty specimens were prepared from each brick sample, ten of them for dry conditions and the other ten for saturated conditions. The Vp measurements of the specimens were performed in accordance with the ISRM [29]. The specimens had a diameter (D) of 44 mm and a length (L) of 88 mm. Figure 4 shows some of the specimens used for the Vp tests. To carry out the tests, the specimens were fitted between the transmitter and the receiver of the Vp device, and the pulse travel time (t) through the transducers was recorded. The Vp values of the specimens were calculated according to the following equation:

(6) $$Vp=\frac{L}{t}$$

## 3. Results and Discussion

### 3.1. Mineralogical Composition and Porosity of the Samples

**Figure 5:**
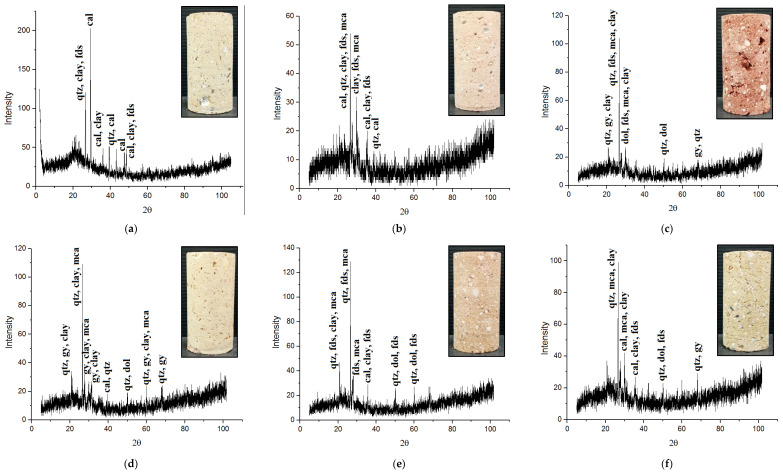
XRD patterns of the samples: (**a**) brick 1, (**b**) brick 2, (**c**) brick 3, (**d**) brick 4, (**e**) brick 5, and (**f**) brick 6. (**qtz**: quartz, **fds**: feldspar, **mca**: mica, **cal**: calcite, **dol**: dolomite, **gy**: gypsum, **clay**: clay).

The XRD patterns of the samples are shown in Figure 5. According to the analysis of the XRD patterns, the types of the constituent minerals of the samples were determined. As can be seen from Table 1, components existing in the samples had a wide range of various minerals including quartz, feldspar, mica, calcite, dolomite, gypsum, chlorite, kaolinite, montmorillonite, and illite.

**Figure 6:**
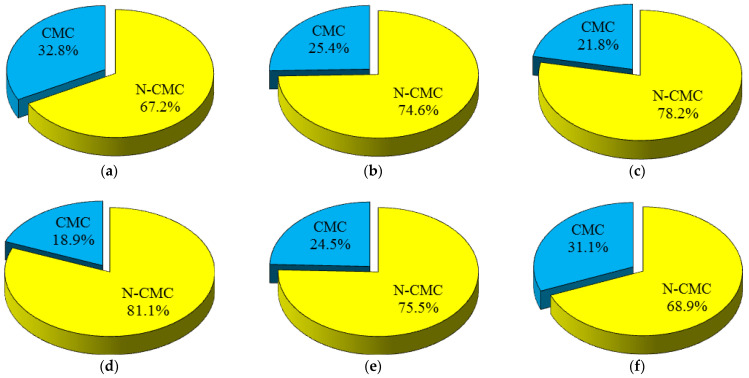
Clay and nonclay mineral content of the samples (CM and N-CM, respectively): (**a**) brick 1, (**b**) brick 2, (**c**) brick 3, (**d**) brick 4, (**e**) brick 5, and (**f**) brick 6.

Clay minerals (i.e., chlorite, kaolinite, montmorillonite, and illite) are highly susceptible to water absorption [28,32]. Any changes in the water content of construction materials containing clay minerals play a critical role in these materials’ engineering behavior. A minor modification in the water content can be associated with a significant integrity loss of the physical and strength characteristics of the construction materials [18,23,26] and therefore can affect their durability against environmental deterioration processes such as freeze–thaw, salt crystallization, and acid corrosion [5,33,34]. With regard to the importance of clay minerals in the engineering behavior of the construction materials, the constituent minerals of the samples were categorized into two groups including clay minerals (CM) and nonclay minerals (N-CM). Based on the data presented in Figure 6, the CM of the samples varied from 18.9 (for brick 4) to 32.8% (for brick 1). Although the CM had lower values compared to the N-CM (ranging from 67.2 to 81.1%), these values were significant and could play a critical role on the UCS, BTS, and Vp after the saturation of the samples.

The n is an important parameter concerning clay bricks due to its effect on characteristics such as chemical reactivity and mechanical strength, effectively affecting a brick’s quality and durability, which increases with decreasing values of n [35]. Commonly, clay bricks exhibit high n values, ranging between 15% and more than 40% [36,37,38,39]. According to Table 2, the brick samples in the present study had an n between 14.67 and 28.13%. A higher n corresponds to higher water absorption of the sample during its saturation. The higher water absorption of a sample can lead to its more severe integrity loss. Therefore, n could be used as an index physical characteristic in the assessment of changes in the UCS, BTS, and Vp of the samples after their saturation. The quantitative–qualitative classification proposed by the IAEG [40] was utilized for the samples. Based on this classification, as shown in Table 2, brick 4 is categorized as a construction material with medium n (5–15%), and the other brick samples fall into the class of high n (15–30%).

### 3.2. Strength Characteristics and Ultrasonic Wave Velocity of the Samples

**Figure 7:**
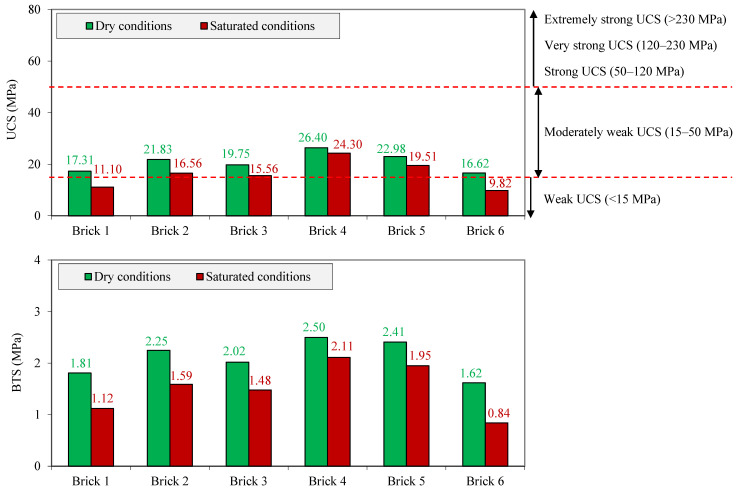

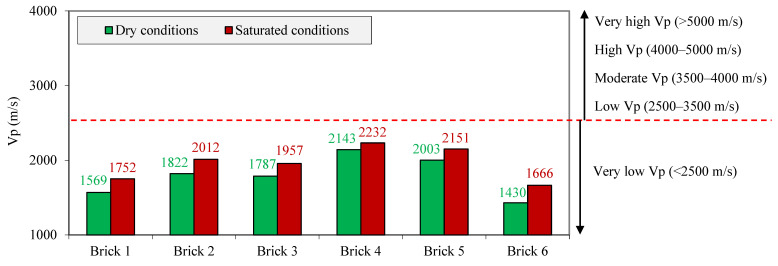
UCS, BTS, and Vp of the samples and their classifications based on IAEG [40].

The values of the UCS, BTS, and Vp of the samples under dry and saturated conditions are given in Figure 7. It can be seen that the UCS of the samples under dry conditions varied from 16.62 to 26.40 MPa, while it was between 9.82 and 24.30 MPa under saturated conditions. These results indicate that for all samples, the UCS values under saturated conditions were lower than those obtained under dry conditions. Under both dry and saturated conditions, the lowest and highest UCS values were obtained for brick 6 and brick 4, respectively. The UCS value is strongly influenced by the characteristics of the raw material as well as by the production process of the brick. In addition, the mineral type and content and the amount of n and Sr also play an important role in the UCS value [35]. A review of the literature showed that depending on the brick’s age, old bricks exhibit a wide range of UCS values, from about 1.5 to 32 MPa and up to 50 MPa [37,41,42]. Currently, with the advancement of technology and new methods in the brick production process as well as the variety of additives, the bricks’ UCS has reached higher values. However, comparing the range of UCS values of the bricks investigated in the present study with the values obtained in previous studies shows an acceptable agreement between the results (Table 3) [43,44,45,46,47].

The samples were classified according to their UCS values using the classification suggested by the IAEG [40]. In this classification, construction materials are categorized into five strength classes including weak (UCS < 15 MPa), moderately weak (UCS 15–50 MPa), strong (50–120 MPa), very strong (120–230 MPa), and extremely strong (>230 MPa). Figure 7 shows that all samples under dry conditions were classified as bricks with moderately weak strength (UCS 15–50 MPa). For the samples under saturated conditions, bricks 2, 3, 4, and 5 were still in the class of moderately weak strength, while bricks 1 and 6, with a one-rank loss compared to dry conditions, fell into the class of construction materials with weak strength (UCS < 15 MPa).

As another index strength characteristic of construction materials, the BTS of the samples under dry conditions had values between 1.62 and 2.50 MPa; in contrast, these values for saturated samples varied from 0.84 to 2.11 MPa. As shown in Figure 7, for all samples, the BTS under saturated conditions showed lower values compared to dry conditions. Among the samples, brick 6 and brick 4 had the lowest and highest BTS values, respectively, under both dry and saturated conditions. The BTS of brick 6 under dry and saturated conditions was equal to 1.62 and 0.82 MPa, respectively, whereas these values were 2.50 and 2.11 MPa for brick 4, respectively. 

The BTS is often reported as a percentage of the corresponding UCS, usually between 3 and 10% and sometimes up to 13.5% [35]. In the study performed by Vatani Oskouei et al. [51], a BTS of 0.04 MPa, equivalent to 0.6% of the UCS value, was obtained for a clay brick sample. Baronio and Binda [55] found a BTS equal to 5.5 MPa, which corresponds to 5.0–6.5% of the respective UCS. In the studies of Binda et al. [56,57], they reported a BTS between 0.1 and 2.6 MPa for bricks from the belltower of the Cathedral of Cremona, Italy, representing 1 and 10% of the respective UCS. However, in the present study, a BTS equivalent to 9.5–10.5% and 8.6–10.1% of the UCS was obtained for samples under dry and saturated conditions, respectively.

The decrease in the strength of the samples (i.e., UCS and BTS) after saturation is due to the softening and lubricating effects of water. Previous studies revealed that due to the presence of clay minerals in some construction materials, such materials are highly susceptible to deterioration when exposed to water [32,58,59]. Since clay minerals are a significant part of the constituent components of the samples (CM 18.9–32.8%) (Figure 6), they make the samples susceptible to the softening and lubricating effects of water, thereby decreasing the samples’ UCS and BTS. The softening mechanism of water is relatively complicated. During the saturation of the samples, water can easily enter the pore spaces, especially in brick samples with a higher CM because clay minerals possess a large specific surface and strong hydrophilicity. Water commonly exists in clay minerals in two forms: hydration water and interlayer water. Hydration water accumulates on the surface of clay minerals, and interlayer water is strongly associated with interlayer cations and exists within the interlayer space [60]. When clay minerals come into contact with water, the water absorbed by them is mainly in the form of hydration water [25]. The bonding between the clay grains is intensely weak, and the clay interlayer can be separated by strongly polarized water molecules with oxygen bonds [61]. As a result, the water will increase the space between layers and cause the samples to swell and soften [62]. In addition to the softening mechanism of water, the lubrication role of water also has a significant effect on the integrity loss of the samples’ UCS and BTS. During the process of sample saturation, water enters the pore spaces of the samples and lubricates the clay minerals. Lubrication occurs when the surface of the clay grains becomes wet and causes the mobility of the absorbed film to increase due to the increased thickness and greater hydration and dissociation of the surface ions [63]. Therefore, the lubrication action of water reduces the internal friction of the sample matrix because water molecules can occupy space within clay layers [25].

According to the literature, a good agreement is observed between the results of the present study and the findings of previous studies conducted by various researchers. Studies of Karakul and Ulusay [23], Khajevand [26], Vasarhelyi [64], and Hamzaban et al. [65] revealed that the UCS and BTS values of construction materials under saturated conditions are lower than those obtained under dry conditions. In any case, by examining the findings of the previous studies and the present study, it should be noted that the UCS reduction and the BTS reduction from saturation of the studied samples exhibit different degrees of sensitivity against the softening and lubricating effects of water.

The Vp results of the samples are presented in Figure 7. It can be seen that the Vp values of the samples under dry conditions varied from 1430 to 2143 m/s. However, the Vp of the saturated samples showed values between 1666 and 2232 m/s. Based on previous studies, the bricks have a wide variation in their constituent mineral types and contents, physical characteristics (especially porosity), and pore size distribution [53], which strongly affect the Vp. As a result, Vp values of the bricks vary in a wide range. However, Vp values of bricks in the present study and those obtained by various researchers are almost consistent with each other (Table 3). 

According to Figure 7, the findings revealed an increase from 89 to 236 m/s in Vp of the samples (for brick 4 and brick 6, respectively) after their saturation. Comparing the findings of the present study with those obtained in previous studies shows a good agreement regarding the increased Vp of the construction materials after their saturation. For example, laboratory studies of Abdi et al. [18], Kahraman [20], and Zalooli et al. [66] on dry and saturated samples revealed a Vp difference of 1200–2400 m/s for various rock types, 330–355 m/s for sandstone, and 320–840 m/s for travertine, respectively. To gain better insight into the reason for the difference between the Vp values of the samples under dry and saturated conditions, the n of the samples can be used, as it is an important factor affecting the change trend of the Vp. A brick with a higher n provides more pore space within the test sample, which is filled with distilled water after saturation. Therefore, it can be expected that the higher the brick’s n, the greater the difference between its Vp values under dry and saturated conditions.

According to the data presented in Figure 7, the UCS, BTS, and Vp values showed changes with different rates when samples were saturated. Here, Equation (7) was used to assess the change rate (CR) of the UCS, BTS, and Vp of the samples after their saturation, as follows:

(7) $${CR}_{\left(UCS,BTS,or\;Vp\right)}=\frac{{{UCS,BTS,or\;Vp}_{\left(saturated\;conditions\right)}-UCS,BTS,or\;Vp}_{\left(dry\;conditions\right)}}{{UCS,BTS,or\;Vp}_{\left(dry\;conditions\right)}}\times 100$$

**Figure 8:**
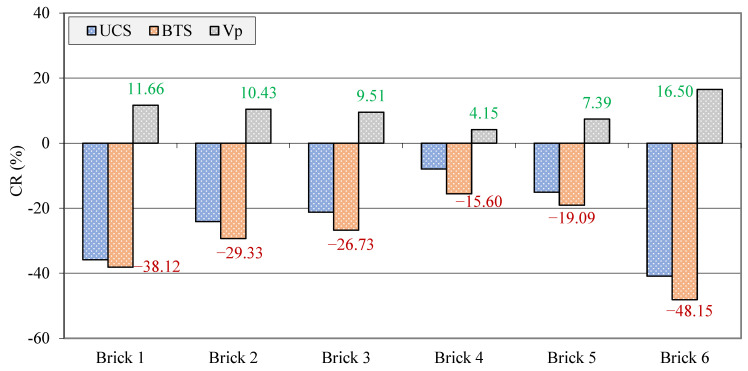
Change rate (CR) of the UCS, BTS, and Vp after saturation of the samples.

The CR values of the UCS, BTS, and Vp calculated using Equation (7) are given in Figure 8. The negative and positive signs of the CR indicate a decrease in the UCS and BTS and an increase in the Vp, respectively. The UCS, BTS, and Vp of the samples had CR values ranging from −7.95 to −40.91%, −15.60 to −48.15%, and 4.15 to 16.50%, respectively. These values revealed that after the saturation of the samples, the UCS and BTS were affected with the highest and Vp with the lowest intensities, respectively. The changes in the UCS, BTS, and Vp values with the saturation of the samples are in good agreement with previous studies, such as those that conducted by Wong and Jong [16], Karakul [17], Torok and Vasarhelyi [21], and Huang and Yu [67]; however, the change rates of the aforementioned characteristics have significant differences with each other. These differences can be attributed to the type of the construction materials, mineralogical composition, textural characteristics, porosity, etc.

The ratio of the saturated-to-dry strength (sat–dry_(strength)_) is known as an index quantity measure to assess the durability of the construction materials against environmental deterioration processes such as wetting–drying cycles [22,66]. Regarding this connection, a durability classification for construction materials was suggested by Cobanoglu and Celik [22] based on the ratio of sat–dry_(strength)_. Equation (8) was used to calculate the sat–dry_(strength)_ of the samples as follows:

(8) $${Sat-dry}_{\left(UCS\;or\;BTS\right)}=\frac{{Saturated}_{UCS\;or\;BTS}}{{Dry}_{UCS\;or\;BTS}}$$

where sat–dry_(UCS or BTS)_ is the ratio of the saturated-to-dry UCS or BTS of the samples. 

**Figure 9:**
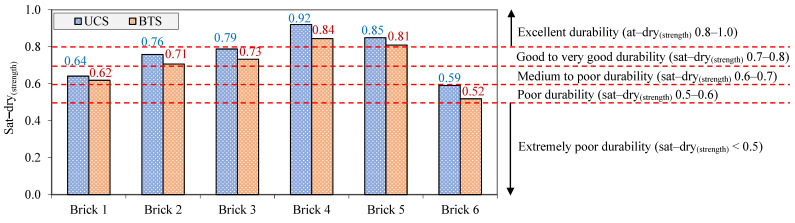
Durability classification of the samples based on the sat–dry ratio of their UCS and BTS [22].

**Figure 10:**
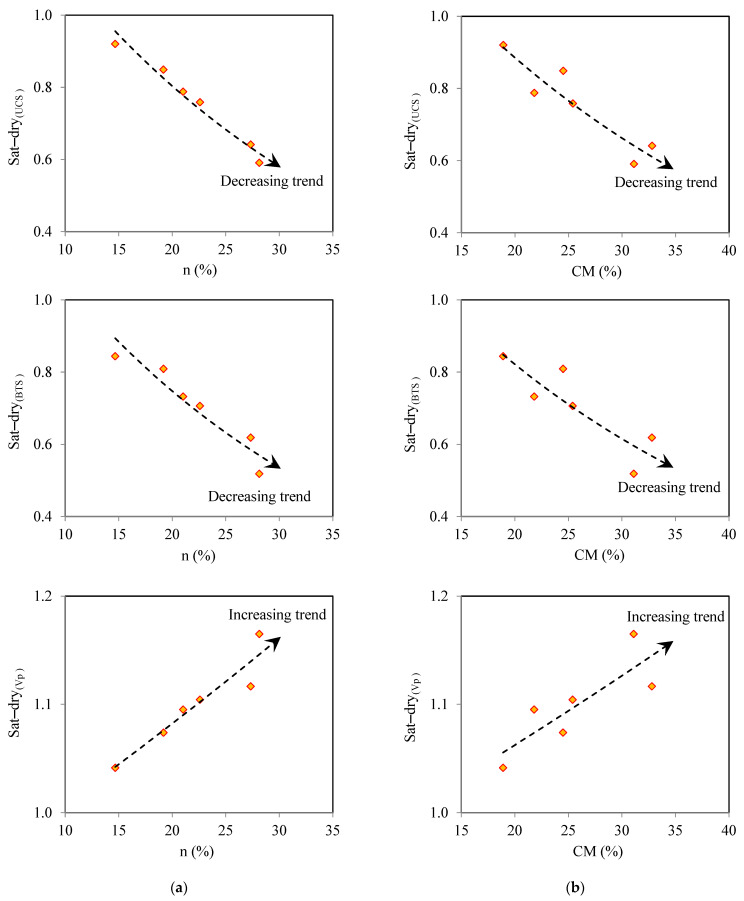
Sat–dry of UCS, BTS, and Vp versus (**a**) n, and (**b**) CM.

Values of the sat–dry_(UCS)_ and sat–dry_(BTS)_ of the studied samples and their durability classes are shown in Figure 9. This figure shows that brick 6 and brick 1 are construction materials in the durability classes of poor and medium to poor, respectively; furthermore, a good to very good durability was obtained for bricks 2 and 3. Finally, the highest durability among the samples belonged to bricks 4 and 5, with an excellent durability. These results indicate that the samples had different durabilities against the softening and lubricating effects of water after their saturation. It is well-known that n and CM are critical parameters controlling the durability behavior of construction materials against the softening and lubricating effects of water [28,32,65]. In order to assess the roles of n and CM on the samples’ durability, the relationships of these parameters with sat-dry_(UCS)_, sat-dry_(BTS)_, and sat-dry_(Vp)_ were investigated. It can be seen from Figure 10 that there are decreasing and increasing trends for sat-dry_(UCS)_, sat-dry_(BTS)_, and sat-dry_(Vp)_, respectively, with increasing n and CM in the samples. In fact, the findings revealed that samples with higher n and CM had lower sat-dry_(UCS)_ and sat-dry_(BTS)_ and higher sat-dry_(Vp)_, indicating their lower durability against the softening and lubricating effects of water. 

### 3.3. Correlating the Strength Characteristics with Ultrasonic Wave Velocity

**Figure 11:**
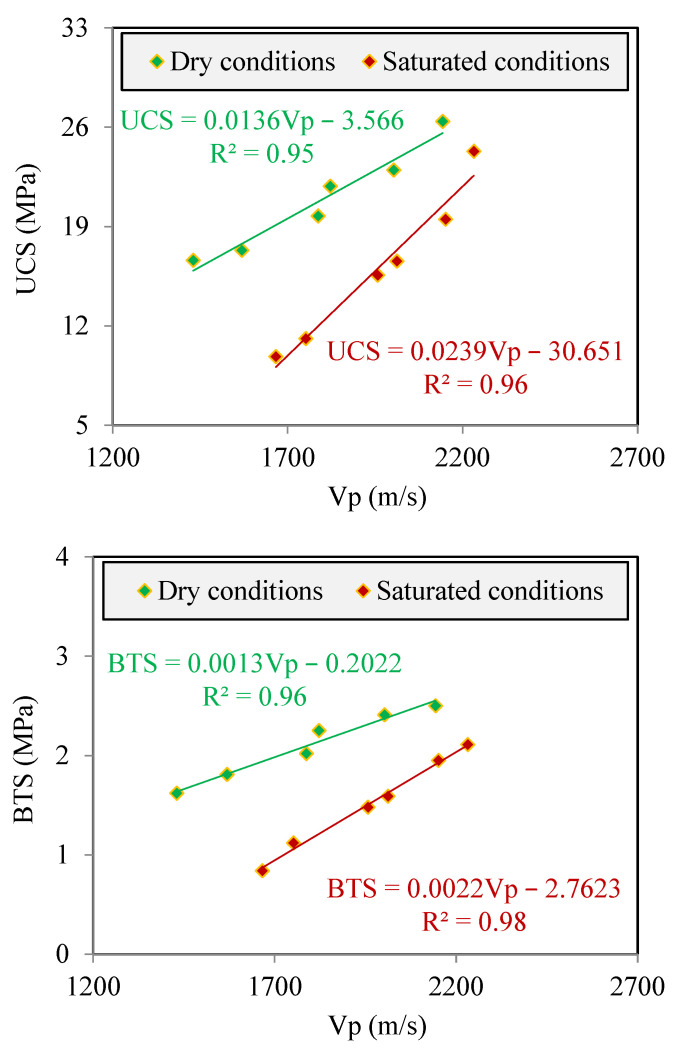
Correlations of the UCS and BTS with the Vp of the samples under dry and saturated conditions.

Correlations of the UCS and BTS with the Vp were investigated using simple regression analyses. The data presented in Figure 7 were used for this purpose. The results of the regression analyses are shown in Figure 11. In these analyses, values of the UCS and BTS are considered as independent and Vp as dependent variables, respectively. Figure 11 shows that there are linear correlations between UCS and Vp under both dry and saturated conditions with good coefficients of determination (R^2^) of 0.95 and 0.96, respectively, as follows:

(9) $$UCS=0.0136Vp-3.566\;\;\;\;\;\;\;R^{2}=0.95\;\;\;\;\;\;\text{for dry conditions}$$

(10) $$UCS=0.0239Vp-30.651\;\;\;\;\;\;\;R^{2}=0.96\;\;\;\;\;\;\text{for saturated conditions}$$

Based on the plots shown in Figure 11, good linear correlations with R^2^ equal to 0.96 and 0.98 were obtained between BTS and Vp under dry and saturated conditions, respectively. Correlation equations between BTS and Vp are as below:

(11) $$BTS=0.0013Vp-0.2022\;\;\;\;\;\;\;R^{2}=0.96\;\;\;\;\;\;\text{for dry conditions}$$

(12) $$BTS=0.0022Vp-2.7623\;\;\;\;\;\;R^{2}=0.98\;\;\;\;\;\;\text{for saturated conditions}$$

As can be seen from the correlation equations developed in Figure 11, an increasing trend in UCS and BTS with increasing Vp can be observed for the samples under both dry and saturated conditions. Based on a review of the literature, researchers such as Jamshidi et al. [12], Abdi et al. [18], and Karakul and Ulusay [23] also showed positive correlations of UCS and BTS with Vp in various construction materials. Although there is a very good agreement of the results of the present study with previous studies regarding the positive trend of correlations of UCS and BTS with Vp, the R^2^ values obtained from the correlation equations in some cases have differences from low to high. A difference in the R^2^ values can be attributed to the test sample type; sample conditions (i.e., dry or saturated); petrographical characteristics of the sample including minerals type and content and textural features; physical characteristics of the sample (such as water content and porosity); and range of UCS, BTS, and Vp of the samples.

The experimental results showed that there are strong positive correlations between the Vp and the UCS and BTS of the studied clay bricks (Figure 11). The reason for the positive correlation between these parameters is as follows. Pores play a significant role in the structural integrity of construction materials, especially bricks. Structural integrity is usually evaluated using the porosity (n), which is known as one of the important physical characteristics of bricks. A brick sample with a lower n will have stronger structural integrity between its constituent components and, as a result, will show more resistance to external forces. On the other hand, the ultrasonic wave velocity in a solid such as a mineral is faster than that in air, which occupies the pore space of bricks. Therefore, the ultrasonic wave velocity will be higher in a brick with a lower n (i.e., less pore space). From the abovementioned discussions, it can be concluded that both strength and ultrasonic wave velocity are functions of structural integrity. A greater structural integrity in the brick leads to a higher strength and ultrasonic wave velocity. Consequently, there is a positive direct relationship between strength and ultrasonic wave velocity.

**Figure 12:**
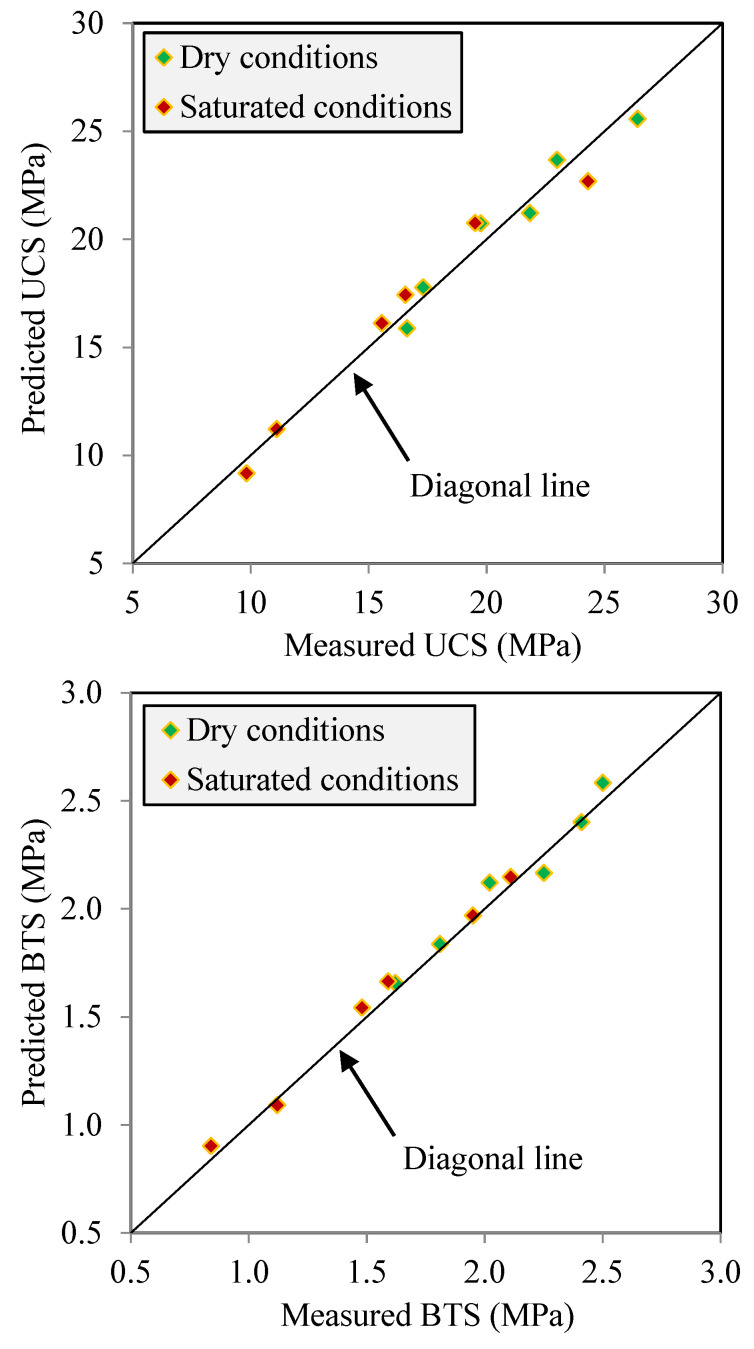
Measured UCS and BTS versus predicted UCS and BTS.

The findings of the regression analyses revealed that there are correlation equations with an acceptable level of the R^2^ ranging from 0.95 to 0.98 between the Vp and the UCS and BTS for samples under both dry and saturated conditions. The high values of the R^2^ indicate that the correlation equations developed in the present study (i.e., Equations (9)–(12)) have good accuracy as simple and practical tools in predicting the UCS and BTS from the Vp. Although R^2^ is a common and widely used quantitative measure in the efficiency assessment of regression analyses [26,68,69], the diagonal line (1:1) was also utilized for a more in-depth investigation of the accuracy degree of the correlation equations. For this, the measured UCS and BTS values were plotted versus those that were predicted using Equations (9)–(12) (Figure 12). The data points closer to the diagonal line indicate a higher accuracy of the correlation equation in predicting the UCS and BTS. In fact, a data point on the diagonal line indicates that the values of the UCS and BTS predicted from correlation equations are exactly what were obtained after performing tests (i.e., the measured values). The error in the predicted values of the UCS and BTS is represented by the distance that each data point has from the diagonal line. According to Figure 10, the data points fit well and are close to the diagonal line; as a result, Equations (9)–(12) are accurate for predicting the UCS and BTS using the Vp for samples under both dry and saturated conditions.

The global usefulness and significance of the predictive equations were investigated using variance analysis for the regression. The F statistics test is one of the most common methods utilized in regression and variance analysis by various researchers [70,71,72]. Table 4 shows the results of the variance analysis for correlation equations. Based on a 95% level of confidence, the tabulated F value with a degree of freedom υ_1_ = 1 and υ_2_ = 4 is 7.71. Since the F values computed for the correlation equations are far larger than the tabulated F values, it is concluded that these equations have good accuracy for predicting the UCS and BTS from the Vp.

For assessing the prediction performance of correlation equations, the statistics indices, including coefficient values accounted for (VAF) and the root mean square error (RMSE), were calculated using Equations (13) and (14), respectively:

(13) $$VAF=\left[1-\frac{var(y-y^{\prime })}{vary}\right]\times 100$$

(14) $$RMSE=\sqrt{\frac{1}{N}\sum_{i=1}^{N}{\left(y-y^{\prime }\right)}^{2}}$$

where y and y′ are the measured and predicted values of the strength characteristics (i.e., UCS or BTS), respectively, ȳ and ȳ′ are the mean values of y and y′, respectively, and N is the number of the dataset. 

A correlation equation is excellent for predicting the unknown variable from the one that is known (in this study: UCS and BTS, and Vp, respectively) if the VAF = 100% and RMSE = 0. Values of these indices for Equations (9–12) were calculated and are given in Table 4. The VAF and RMSE values of these equations are in the range of 95.19 to 99.38% and 0.04 to 0.75, respectively. All these values are at very good levels, indicating the high performance of correlation equations in predicting the UCS and BTS using Vp for samples under both dry and saturated conditions. As a result, correlation equations are efficient and accurate in the indirect assessment of the UCS and BTS of clay bricks when measured data of these characteristics are not available.

In this section, the validity of the predictive equations was evaluated using the data published by Noor-E-Khuda and Albermani [39]. These researchers experimentally investigated the UCS and Vp of four different clay bricks collected from recently demolished old building sites in Perth, Western Australia. The measured values of the UCS and Vp in the study of Noor-E-Khuda and Albermani [39] are summarized in Table 5. The UCS of the brick samples in the study of Noor-E-Khuda and Albermani [39] were predicted from their Vp through Equation (9) developed in the present study. The prediction error of the UCS was calculated using Equation (15):

(15) $$Prediction\;error\;\left(\%\right)=\frac{{UCS}_{m}-{UCS}_{p}}{{UCS}_{m}}\times 100$$

where UCS_m_ and UCS_p_ are values of the measured and predicted UCS, respectively.

According to the results given in Table 5, the prediction errors of the UCS of the brick samples are between 17.3 and 84.4%. The findings showed that the prediction error for one of the brick samples (W-series) (17.3%) is at an acceptable level, whereas other samples including H-series, O-series, and S-series had a higher prediction error at 60.4%. The difference in the prediction error and, thus, the performance of the predictive equations can be attributed to the heterogeneity of clay bricks due to their porous nature, wide variety of mineralogical composition, and physicomechanical properties, which cause the bricks to behave differently. As a conclusion, the predictive equations developed in the present study can be simple, fast, low-cost tools for predicting the strength characteristics using the Vp of clay bricks in other regions of the world, provided that the ranges of their mineralogical compositions and physicomechanical characteristics are similar to the bricks investigated in the present study. However, further studies in this regard need to be undertaken by researchers.

## 4. Conclusions

Deterioration of construction materials used in buildings under environmental weathering factors is one of the challenging issues for researchers. Detecting the beginning of the deterioration process in construction materials can be of great help in order to apply some preventive measures against progressive deterioration. Since the durability of construction materials (i.e., resistance to deterioration) is significantly affected by their strength characteristics, one of the solutions for detecting the beginning of the deterioration process can be monitoring the strength characteristics of construction materials over time. Due to the nature of strength characteristics tests, in some cases, it is not possible to directly measure these characteristics. As an alternative method, ultrasonic wave velocity as a nondestructive, low-cost, fast, and cheap test can be applied for the indirect assessment of the strength characteristics of construction materials. 

In the present study, six different samples of clay bricks were collected. The test specimens from bricks were prepared for both dry and saturated conditions. The mineralogical composition, porosity (n), strength characteristics [including uniaxial compressive strength (UCS) and Brazilian tensile strength (BTS)], and P-wave velocity (Vp) of the brick samples were determined. After an in-depth analysis of the data, the following results were obtained:

- After the saturation of the samples, significant reductions occurred in the UCS and BTS ranging from −7.95 to −40.91% and −15.60 to −48.15%, respectively, whereas an increase was observed for the Vp from 4.15 to 16.50%. The main reasons for the changes in the UCS and BTS and in the Vp were the softening and lubricating effects of water on the saturated samples.
- The change level of the UCS, BTS, and Vp after saturation of the samples was strongly affected by the n and clay minerals (CM). An increasing trend in the change intensity of the UCS, BTS, and Vp with the increasing n and CM of the sample was obtained.
- Based on values of the saturated-to-dry UCS and BTS, samples with lower n and CM have the highest durability against the softening and lubricating effects of water. An increase in the n and CM will lead to more intense integrity loss of the samples after their saturation.
- Good correlation equations with determination coefficients (R^2^) varying from 0.95 to 0.98 were obtained between the Vp and the UCS and BTS. The significance and performance of the equations were evaluated using a diagonal line, variance analysis, and statistics indices including coefficient values accounted for (VAF) and the root mean square error (RMSE). The results indicated that correlation equations are at a good accuracy level for predicting the UCS and BTS from the Vp for samples under both dry and saturated conditions.
- The predictive equations of the UCS and BTS from the Vp can be used for clay bricks from other buildings in the world with ranges of UCS, BTS, and Vp similar to the samples in the present study. As a result, in some cases where preparing test samples from a building is not possible for the direct measurement of the UCS and BTS, these equations can be used to avoid performing UCS and BTS tests. Such predictive equations can be developed for various bricks with a wide range of mineralogical compositions and physicomechanical characteristics. Therefore, further studies in this regard need to be undertaken by researchers in the future.
- It must be noted that clay bricks are notoriously variable and heterogeneous in their UCS, BTS, and Vp, which depend on the nature of their porous media such as the size distribution and shape of the pores and internal structure. Therefore, predictive equations for the UCS and BTS can be developed for various bricks with a wide range of mineralogical compositions and physicomechanical characteristics. In this regard, further studies need to be performed by researchers in the future.

## Figures and Tables

**Table 1:** Mineralogical composition of the samples.

Sample Code		Nonclay Minerals (N-CM)							Clay Minerals (CM)			
		Quartz	Feldspar	Mica	Calcite	Dolomite	Gypsum		Chlorite	Kaolinite	Montmorillonite	Illite
Brick 1		√	√		√				√	√	√	
Brick 2		√	√	√	√							√
Brick 3		√	√	√		√	√		√			√
Brick 4		√		√	√	√	√		√			√
Brick 5		√	√	√		√				√		√
Brick 6		√	√	√	√	√	√		√		√	√

**Table 2:** Samples’ classification based on their n values [40].

Sample Code	n (%)	* n Description				
		VL	L	M	H	VH
Brick 1	27.32				√	
Brick 2	22.59				√	
Brick 3	21.02				√	
Brick 4	14.67			√		
Brick 5	19.18				√	
Brick 6	28.13				√	

* **VL**: very low (n < 1%), **L**: low (n = 1–5%), **M**: medium (n = 5–15%), **H**: high (n = 15–30%), and **VH**: very high (n > 30%).

**Table 3:** UCS and Vp of clay bricks in some previous studies.

Researcher/s	UCS (MPa)	Vp (m/s)
Crespo-Lopez et al. [1]	6.4–72.6	1260–4625
Noor-E-Khuda and Albermani [39]	20.43–45.15	2507–3162
Koroth [43]	24.2–143.5	2201–4383
Grubesa et al. [46]	~27.0–56.0	No data
Azam et al. [47]	~5.0–24.0	~1380–3300
Karaman et al. [48]	8.5–31.4	No data
Johari et al. [49]	2.5–89.5	No data
Abbas et al. [50]	~23.0	No data
Vatani Oskouei et al. [51]	4.4	No data
Araujo et al. [52]	2.9–11.9	586–3520
Chapagain et al. [53]	~3.2–10.5	No data
Rasanen et al. [54]	~18.0–61.5	~1300–3500
The present study	9.82–26.40	1430–2232

**Table 4:** Results of statistical tests for the correlation equations.

Equation No.	Correlation Equation	Sample Conditions	VAF (%)	RMSE	F Value		F Sig.
					Computed	Tabulated	
(9)	$UCS=0.0136V_{p}-3.566$	Dry	95.19	0.57	79.08	7.71	0.001
(10)	$UCS=0.0239V_{p}-30.651$	Saturated	96.10	0.75	98.52	7.71	0.001
(11)	$BTS=0.0013V_{p}-0.2022$	Dry	96.29	0.05	104.15	7.71	0.001
(12)	$BTS=0.0022V_{p}-2.7623$	Saturated	99.38	0.04	647.78	7.71	0.000

**Table 5:** Measured values of Vp and UCS by Noor-E-Khuda and Albermani [39] and UCS predicted from Equation (9) developed in the present study.

Brick Code	Brick Age (year)	Measured Parameter		Predictive Equation	Predicted Parameter	^a^ Error (%)
		Vp (m/s)	UCS (MPa)		UCS (MPa)	
H-series	25–30	2703	18.00	$UCS=0.0136V_{p}-3.566$	33.19	−84.4
W-series	25–30	2723	28.54	$UCS=0.0136V_{p}-3.566$	33.47	−17.3
O-series	45–50	2340	16.18	$UCS=0.0136V_{p}-3.566$	28.26	−74.6
S-series	45–50	2385	18.00	$UCS=0.0136V_{p}-3.566$	28.87	−60.4

^a^ The negative sign indicates that the predicted value was higher than the measured value.

## Data Availability

Data are contained within the article.
